# Genome-wide cloning, identification, classification and functional analysis of cotton heat shock transcription factors in cotton (*Gossypium hirsutum*)

**DOI:** 10.1186/1471-2164-15-961

**Published:** 2014-11-06

**Authors:** Jun Wang, Na Sun, Ting Deng, Lida Zhang, Kaijing Zuo

**Affiliations:** Plant Biotechnology Research Center, School of Agriculture and Biology, Shanghai Jiao Tong University, Shanghai, 200240 China

**Keywords:** Heat shock transcriptional factors, *Gossypium hirsutum*, Heat stress, qRT-PCR, Fiber development

## Abstract

**Background:**

Heat shock transcriptional factors (Hsfs) play important roles in the processes of biotic and abiotic stresses as well as in plant development. Cotton (*Gossypium hirsutum*, 2n = 4x = (AD)_2_ = 52) is an important crop for natural fiber production. Due to continuous high temperature and intermittent drought, heat stress is becoming a handicap to improve cotton yield and lint quality. Recently, the related wild diploid species *Gossypium raimondii* genome (2n = 2x = (D_5_)_2_ = 26) has been fully sequenced. In order to analyze the functions of different Hsfs at the genome-wide level, detailed characterization and analysis of the *Hsf* gene family in *G. hirsutum* is indispensable.

**Results:**

EST assembly and genome-wide analyses were applied to clone and identify heat shock transcription factor (*Hsf*) genes in Upland cotton (*GhHsf*). Forty *GhHsf* genes were cloned, identified and classified into three main classes (A, B and C) according to the characteristics of their domains. Analysis of gene duplications showed that *GhHsfs* have occurred more frequently than reported in plant genomes such as *Arabidopsis* and *Populus*. Quantitative real-time PCR (qRT-PCR) showed that all *GhHsf* transcripts are expressed in most cotton plant tissues including roots, stems, leaves and developing fibers, and abundantly in developing ovules. Three expression patterns were confirmed in *GhHsf*s when cotton plants were exposed to high temperature for 1 h. *GhHsf39* exhibited the most immediate response to heat shock. Comparative analysis of *Hsf*s expression differences between the wild-type and fiberless mutant suggested that *Hsf*s are involved in fiber development.

**Conclusions:**

Comparative genome analysis showed that Upland cotton D-subgenome contains 40 *Hsf* members, and that the whole genome of Upland cotton contains more than 80 *Hsf* genes due to genome duplication. The expression patterns in different tissues in response to heat shock showed that *GhHsf*s are important for heat stress as well as fiber development. These results provide an improved understanding of the roles of the *Hsf* gene family during stress responses and fiber development.

**Electronic supplementary material:**

The online version of this article (doi:10.1186/1471-2164-15-961) contains supplementary material, which is available to authorized users.

## Background

Plants have developed complex transcriptional systems that are responsive to different environmental stresses [1]. WRKY [2], MYB [3], AP2/ERF [4], NAC [5], bZip [6] and heat shock transcription factors (Hsfs) [7, 8] participate in these complex and overlapping processes. These transcription factors are activated and regulate the expression of thousands of genes to sustain plant growth under unfavorable conditions [9, 10]. Among these transcription factors, Hsfs have attracted particular interest recently because they are involved in many aspects of protein homeostasis including refolding, assembly and transport of damaged proteins to maintain intracellular protein stability under conditions of stress [7, 8, 11, 12].

The *Hsf* gene was first cloned from fruitfly larvae and exists generally in higher eukaryotes [13]. In contrast to only one to four *Hsf* genes in yeast and animals, more than 52 *Hsf* homologs have been identified in the sequenced *Populus* genome [14]. The diversity and multiplicity of Hsfs in plants may result from gene duplication and functional diversity during the evolution of the genome [14–18]. Hsfs are a type of transcription factor that is characterized by a DNA-binding domain (DBD) and hydrophobic heptad repeat regions (HR-A/B) [19–21]. The DBD domain is a conserved structure, which provides Hsf proteins with the ability to bind heat shock cis-elements [20]. The function of the HR-A/B domain in Hsf proteins allows them to form active homologous trimers [22]. Under a variety of stress conditions, latent Hsfs are assembled into the activated trimeric conformation [23]. The transcription factor complexes then bind to the cis-elements of the promoters of target genes such as *Hsp30*, *70* and *90* to activate their expression [22, 24–27]. Based on structural characteristics and phylogenetic comparisons, plant Hsfs are grouped into three main classes: A, B and C [18, 19]. All of class A and C have an extended HR-A/B region with the insertion of different amino acid residues between the A and B regions (21 amino acid residues for class A and 7 for class C). In contrast to class A and class C Hsfs, the HR-A/B region in class B Hsfs does not contain any insertions. Besides the DBD and HR-A/B domains, the functional modules in Hsfs also contain putative nucleus location signal (NLS), nucleus export signal (NES) and transcriptional activation (AHA) motifs [14, 28, 29]. Sequence comparisons and structural analyses indicate that the combination of an AHA motif and a NES represents the signature domain in class A Hsfs [30]. Although class B and C Hsfs lack AHA motifs and they cannot self-activate, they regulate the expressions of heat shock inducible genes through binding to their cis-elements [14].

It has been shown that Hsfs in plants serve as regulators of tolerance to biotic and abiotic stresses [31–34]. Over-expressed *HsfA1b* in *Arabidopsis thaliana* increases water productivity and harvest index under water-replete and water-limiting conditions [35]. HsfA2 in *Arabidopsis* controls the responses to salt, osmotic stress, anoxia and submergence [36]. *Arabidopsis* HsfA1a was shown to sense heat stress and pH changes directly through binding to *HSP18.2* and *HSP70* promoters [37]. In addition to their roles in stress tolerance, Hsfs also perform key roles in development. *HsfB4* in *Arabidopsis* (also known as *SCZ*) is specifically expressed in the quiescent center, the ground tissue initials and the endodermis and cortex in the postembryonic root. In both *SCZ* deletion and *SCZ* over-expressed plants, asymmetric division required for cell-fate separation is affected, demonstrating that SCZ is a regulator of cell-fate separation [38]. Another *Arabidopsis* Hsf protein, Hsf4, which specifically binds to the cis-element of *TBF1* is required for the induction of immune response genes. Functional analysis and genome-wide expression profiling indicate that TBF1 performs a pivotal role in the transition from growth to pathogen defense [39]. Despite these efforts in *Arabidopsis* and tomato, the functions of most *Hsf* genes in plants have not been identified and characterized, probably due to functional redundancy and limited information about this gene family.

Cotton has been the major resource of natural fiber in recent decades. Sustainable cotton production is challenged by continuous high temperatures, intermittent drought and insufficient water supply [40]. Therefore, improved stress tolerance in cotton cultivars is required to reduce the impact of stress and then increase cotton productivity. *Hsf* genes have been proposed to encode the master regulators of biotic and abiotic stresses as well as different developmental processes in plants [14]. Previous studies have suggested the existence of a large gene family within the tetraploid Upland cotton genome, but limited data characterizing these *Hsf* genes in cotton has been presented [41]. In order to gain a comprehensive image of the molecular and evolutionary characteristics as well as the possible functions of the cotton Hsf family, it is necessary to clone *Hsf* gene families and identify their expression patterns. Recently, the full genome sequence of diploid cotton (*G. raimondii*) has been published [41, 42]. This provides the genomic information required for of complete cloning and annotation of *Hsf* genes.

Here, we report the cloning of the D-subgenome *Hsf* genes in Upland cotton. Analysis of their expression profiles in different organs/tissues and the effects of heat shock conditions were conducted by qRT-PCR. The results of this work provide a foundation for an improved understanding of the functional structures and genomic organization of the *Hsf* gene family in cotton, and will undoubtedly be useful in detailed characterization of gene function.

## Material and methods

Upland cotton (*G. hirsutum* L.) variety Coker 312 was grown in the field at the Shanghai Jiao Tong University in China. When cotton plants were in full bloom (approximately 90 days after planting), different cotton tissues including roots, stems, leaves and developing ovules at different stages were collected and used for RNA and DNA extraction.

In order to clone all members in the Hsf protein family in Upland cotton, *Arabidopsis* Hsf protein sequences were used to search the cotton expressed sequence tags (EST) database (http://www.ncbi.nlm.nih.gov) using tBlastN. All putative ESTs encoding Hsf proteins in Upland cotton were assembled to build the putative cotton *Hsf* sequences. All the putative cotton Hsf proteins were compared with the *Arabidopsis* Hsf protein in BlastP searches with *P*-values less than 0.0001 to check whether the putative *Hsf* gene encoded a full-length Hsf protein. Primers were then designed to amplify the coding sequences of all the predicted cotton *Hsf* genes. The amplified fragments were cloned into *pGEM-T* Easy vector (Takara, Japan) and confirmed by DNA sequencing. In order to avoid generating the chimeric genes during PCR amplification [43], all of cloned *GhHsf* genes were compared with those from diploid cotton (*G. arboum and G. ramondii*) on the genomic level [41, 44], and those genes from A sub-genome were cloned again and revised. Finally, all the sequences encoding Hsf proteins were assigned to the D-genome chromosome. Similarities at the same locus in chromosome pairs were considered to represent alleles. Cotton *Hsf* genes were numbered (1, 2, 3 etc.) according to their localization on the chromosomes.

### Domain and protein structure analysis

The deduced amino acid sequences of cotton Hsf proteins were aligned with the *Arabidopsis* Hsf family using DNAMAN and ClustalX 1.83 [45]. Molecular weight, iso-electric point, functional domains, and amino acid signal peptides of cotton Hsfs were calculated using the ExPASy online servers [46] (http://cn.expasy.org/tools). A neighbor-joining (NJ) tree of Hsf proteins was constructed using the MEGA program (version 5.0) [47]. NJ analysis was performed with the Pairwise Deletion option and the Poisson correction. For statistical reliability, bootstrap analysis was conducted with 1,000 replicates to assess the statistical support for each node.

To analyze the signature domains in Hsf proteins, the cotton Hsf proteins were compared with those from *Arabidopsis* and *Populus* by amino acid alignment using ClustalW (version 1.83). The presence of DBDs and coiled-coil structures were determined using the SMART and MEME programs [48–50]. In order to improve the accuracy of domain analysis, MEME tools were also used to identify putative domain motifs in the full-length amino acid sequences of cotton Hsfs. Visualization of the motifs in the cotton Hsf proteins was performed by using ProSite my domains online (http://prosite.expasy.org/mydomains).

### Gene duplication analysis

Cotton *Hsf* gene duplication during evolution was investigated using MEGA (version 5.0). Evolutionary distances between each *GhHsf* sequence pair were calculated by ClustalW [51]. *Hsf* genes duplication was indicated by (1) shared aligned sequence covering >80% of the longer gene and (2) similarity of the aligned regions >80%.

### Cotton Hsf protein localization

To investigate subcellular localization of cotton Hsf proteins, one protein from each subclass including subclass B (GhHsf3) and subclass C (GhHsf31) was chosen to analysis. Considering the function diversity of subclass A, three Hsfs GhHsf39 (A2), GhHsf25 (A1c) and GhHsf34 (A4a) were also used as the representatives. The coding regions of five cotton *Hsfs* (*GhHsf3, 25, 31, 34 39*) from three classes were cloned into the *pBIB-GFP* vector to generate *pBIB-35S::GhHsfs-GFP::Nos* constructs, To test whether the expression level is changed after heat shock, constructed *pBIB-GFP* vectors containing the promoters and ORFs of *GhHsf34* and *GhHsf39* were also generated. These plasmids were then transformed into *Agrobacterium* strain EHA105. Three-week-old tobacco leaves were infiltrated with *Agrobacterium* according to a reported method [52]. Two to four days later, the subcellular localization of Hsf proteins was analyzed by confocal microscopy (Leica TCS SP5) and the fluorescence intensity was also analyzed after heat shock for one hour. .

### Heat shock treatment and qRT-PCR analysis

Cotton seedcoats were removed and sterilized with 0.1% HgCl_2_, and then grown in pasteurized sand in the greenhouse (light/dark cycle: 14 h at 25°C/10 h at 22°C, respectively; 70% relative humidity). At the five-leaf stage, whole plants were subjected to heat shock treatment. The seedlings were initially treated at 45°C for 1 h, before transfer to normal growth conditions for recovery. Subsequently, at 2 h and 4 h, cotton leaves were collected for total RNA extraction.

Quantitative RT-PCR (qRT-PCR) analysis was performed using the SYBR qRT-PCR kit (Takara, Japan) in a DNA Engine Option 3 System (MJ Research, USA) according to the manufacturers’ instructions. The qRT-PCR reaction contained 0.5 μg of 1^st^ cDNA, 1 U ExTaq, 10 pM dNTPs, 5 pM MgCl_2_ and 10 pM primers. Gene-specific primers (Additional file 1: Table S1) were used to amplify specific regions of different cotton *Hsfs*. The ubiquitin gene [52] was used as the internal control. Transcriptional expression levels were calculated using the comparative _Δ_CT method. Each sample was repeated at least four times, and the amplification results were analyzed by Option 3 software.

## Results

### Cloning and identification Hsf gene families in Upland cotton

To clone *Hsf* family genes in cotton, the amino acids of Hsf encoded proteins were used in a tBlastN search of the NCBI database for EST homologs. All of ESTs from Upland cotton showing 60% similarity to *Arabidopsis Hsf* genes were collected for *Hsf* gene assembly. A total of 43 *Hsf* contigs, containing open reading frames encoding the proteins similar to *Arabidopsis Hsfs*, were assembled and identified. All the putative *Hsf* genes were then analyzed for the presence of HR domains and DBD structures within the encoded proteins [14]. Three contigs without these two structures were discarded and 40 cotton *Hsf* genes were then used as the reference for gene cloning. According to the assembled sequences of the putative cotton *Hsf* genes, 40 independent genes were amplified and sequenced using Upland cotton 1^st^ cDNA from different tissues as the template. All of the cloned genes were confirmed to be from D sub-genome after comparing with their homologues from diploid cotton (*G. arboum and G. ramondii*, Additional file 2: Table S2 and Additional file 3). Following comparison with the predicted *Hsf* genes in the D-genome, all 40 *Hsf* genes were then mapped to the different chromosomes in the D-genome [41], and designated *GhHsf1-40* according to the order of their chromosomal localizations (Table 1). All 40 *Hsfs* were distributed in 13 chromosomes and one linkage group in the cotton D-genome. Only one tandem cluster containing 4 *Hsf* genes was found on chromosome 4.

**Table 1:** **Cloning and identification of cotton** ***Hsf*** **family genes**

Gene	Gene locus in ***G.ramondii***	Amino acids	pI	MW (Da)	Chromosome
***GhHsf1***	Gorai.001G012700	343	5.87	40037.16	1
***GhHsf2***	Gorai.002G135200	332	8.27	37421.34	2
***GhHsf3***	Gorai.003G023500	295	6.05	32506.19	3
***GhHsf4***	Gorai.003G053300	502	6.21	55588	3
***GhHsf5***	Gorai.003G091700	238	8.58	27518.09	3
***GhHsf6***	Gorai.003G160600	310	5.27	34627.59	3
***GhHsf7***	Gorai.003G183900	515	4.78	56720.74	3
***GhHsf8***	Gorai.004G076900	362	5.66	41686.65	4
***GhHsf9***	Gorai.004G208800	311	5.02	34368.2	4
***GhHsf10***	Gorai.004G257000	503	5.53	55394.64	4
***GhHsf11***	Gorai.004G280100	327	6.17	36589.64	4
***GhHsf12***	Gorai.004G284200	496	5.53	56720.6	4
***GhHsf13***	Gorai.005G027500	495	5.43	55737.3	5
***GhHsf14***	Gorai.005G102000	343	8.14	38575.58	5
***GhHsf15***	Gorai.006G087100	191	7.71	22255.34	6
***GhHsf16***	Gorai.006G125000	258	9.32	29765.52	6
***GhHsf17***	Gorai.006G158000	482	4.81	53912.11	6
***GhHsf18***	Gorai.006G224000	477	5.51	53905.72	6
***GhHsf19***	Gorai.006G242400	340	5.80	39491.57	6
***GhHsf20***	Gorai.007G010900	326	6.92	36273.79	7
***GhHsf21***	Gorai.007G033300	479	4.74	52198.91	7
***GhHsf22***	Gorai.007G053900	295	7.67	33372.16	7
***GhHsf23***	Gorai.007G139600	345	5.22	39812.5	7
***GhHsf24***	Gorai.008G170800	357	4.66	41293.15	8
***GhHsf25***	Gorai.008G225200	511	5.10	56197.53	8
***GhHsf26***	Gorai.008G244400	304	5.58	33899.14	8
***GhHsf27***	Gorai.009G024700	350	5.67	39696.82	9
***GhHsf28***	Gorai.009G032300	447	4.96	50753.62	9
***GhHsf29***	Gorai.009G213100	360	8.44	40131.3	9
***GhHsf30***	Gorai.010G020700	313	6.51	35645.86	10
***GhHsf31***	Gorai.010G070900	340	5.47	38603.7	10
***GhHsf32***	Gorai.010G240800	384	5.36	44645.69	10
***GhHsf33***	Gorai.011G027400	221	8.95	25756.83	11
***GhHsf34***	Gorai.011G036400	403	5.08	46024.05	11
***GhHsf35***	Gorai.011G105700	279	6.23	30749.59	11
***GhHsf36***	Gorai.011G168400	357	5.51	41384.46	11
***GhHsf37***	Gorai.012G044200	394	5.53	45002.18	12
***GhHsf38***	Gorai.013G183500	432	5.10	47712.05	13
***GhHsf39***	Gorai.013G220400	380	4.76	42597.48	13
***GhHsf40***	Gorai.N013300	379	4.66	43357.61	13

Cotton *Hsf* genes are listed in order of chromosomal location. Protein indexes include sequenced ID, protein size, iso-electric point (pI) and molecular weight (MW) (pI and MW were calculated online; http://www.expasy.org/).

### Conserved domains and motifs in cotton Hsfs

**Figure 1:**
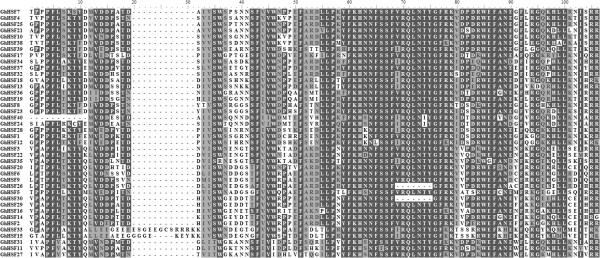
**Multiple sequence alignment analysis of the DBD domains of GhHsf proteins.** Amino acid sequence alignment was performed using BioEdit software.

**Figure 2:**
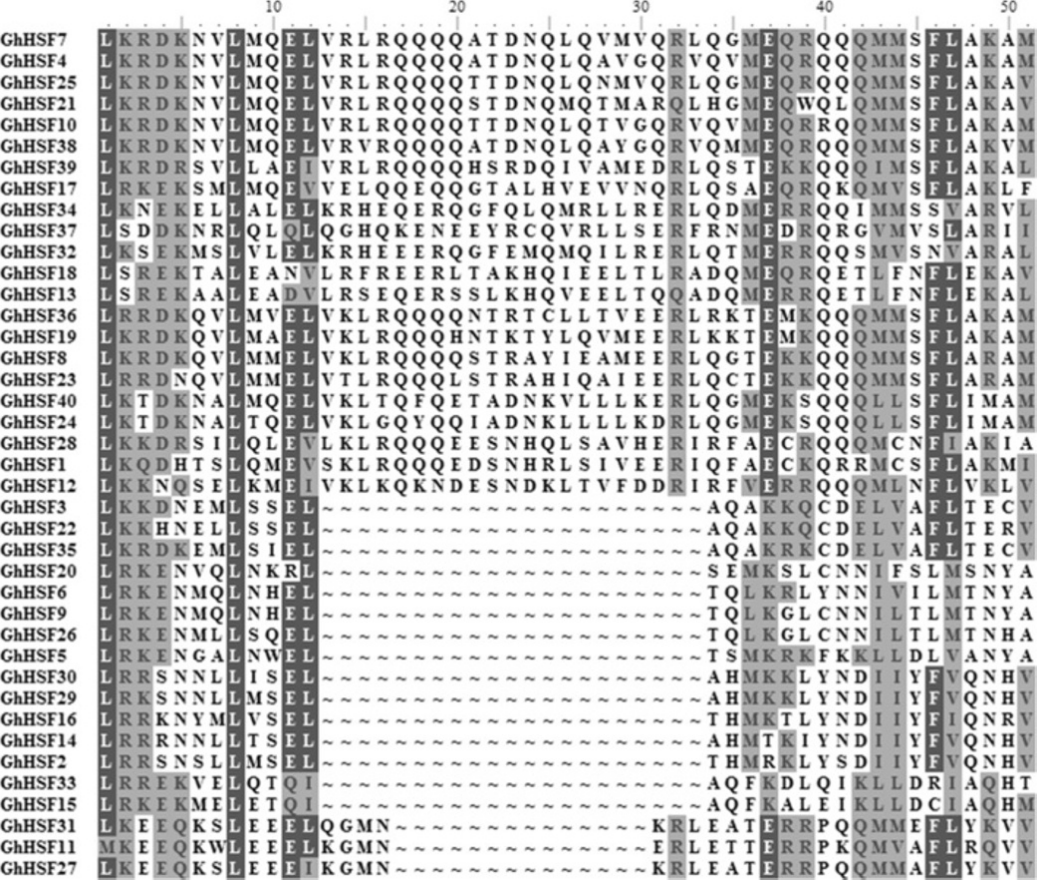
**Multiple sequence alignment of the HR-A/B regions of GhHsf proteins.** The structures between HR-A and HR-B consist of 21 amino acid and 7 amino acid insertions, respectively, for Class A and C. Amino acid sequence alignment was performed using BioEdit software.

**Figure 3:**
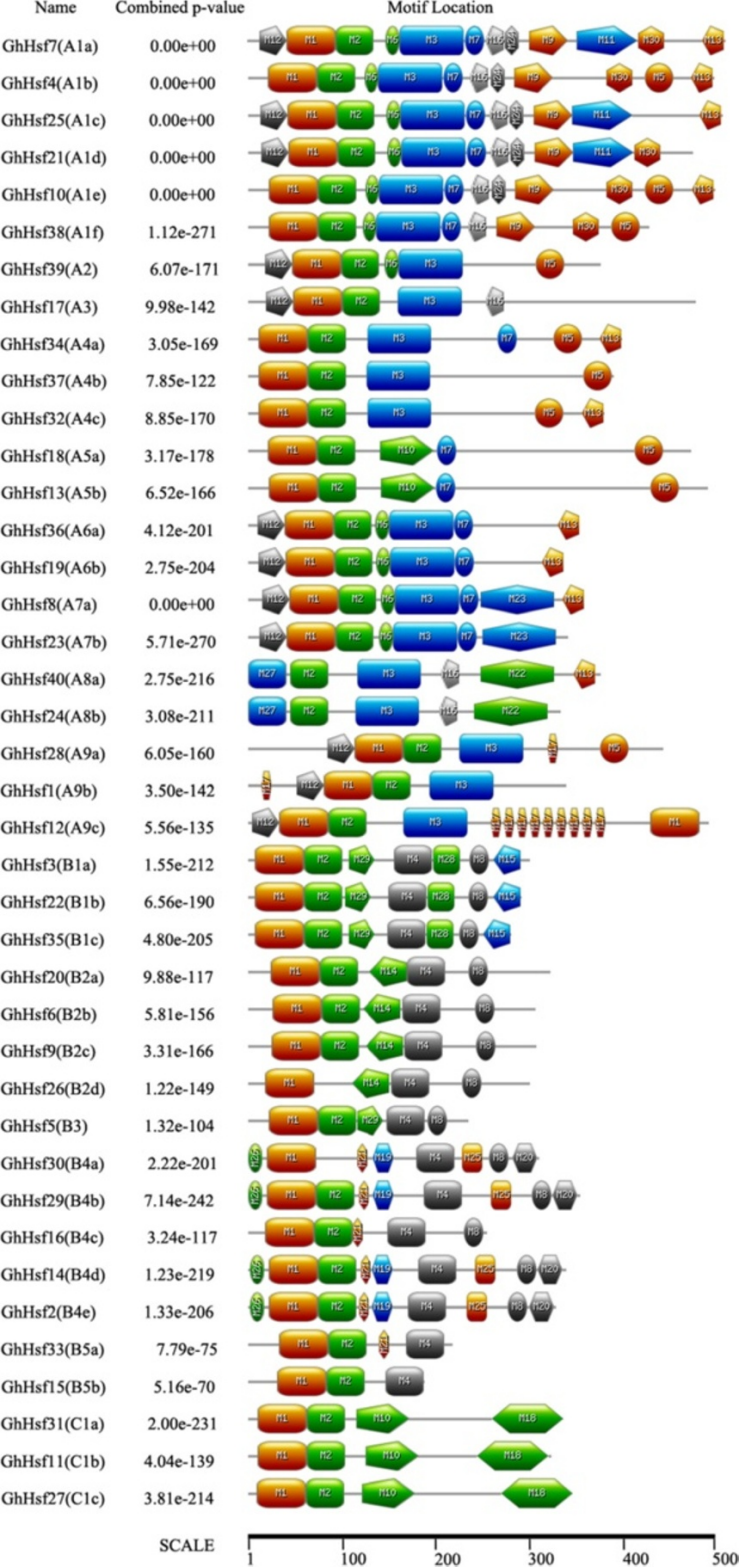
**Motifs in GhHsfs were identified by MEME tools.** The motifs in cotton GhHsfs were analyzed by MEME tools. The results were then downloaded and submitted to http://www.expasy.com to generate the pictures.

The typical Hsf proteins in the plant kingdom contain five conserved domains: DBD, HR-A/B region (also known as the oligomerization domain), NLS and NES motifs and AHA domain. These domains enable Hsf proteins to perform the functions associated with stress tolerance efficiently. All the cotton Hsf proteins were analyzed to detect conserved domain structures online (http://www.expasy.com) and MEME tools (Figures 1, 2 and 3).

The DBD in Hsf proteins is required for specific recognition of the cis-elements of *Hsp* promoters during the transcriptional response to stress. The DBD is composed of an anti-parallel four-stranded β-sheet against three α-helices, forming a compact globular structure. Multiple alignment showed that the highly conserved DBD is located close to the N-terminal in all the cotton Hsf proteins (Table 2, Figure 1), and comprises 83, 94, 102 or 105 amino acid residues. The smallest DBD occurs in GhHsf24 and GhHsf29, 30, 40 that is composed of 83 amino acids, while the largest is in GhHsf33 comprising 105 amino acids. The DBD in the majority of cotton Hsf proteins (34 GhHsf proteins) has 94 amino acids in length, indicating that this domain is highly conserved in GhHsf proteins. Interestingly, while most GhHsfs have a short N-terminal upstream of the DBD (<30 amino acids), GhHsf1 and GhHsf28 contain long N-terminals rich in Ser (80 and 113 amino acids in GhHsf1 and GhHsf28 respectively) (Table 2, Figure 1). The function of this long N-terminal upstream of the DBD domain remains to be determined.

**Table 2:** **Cotton Hsf protein functional domain analysis**

Gene name	Gene locus in ***G.ramondii***	Protein type (A-B-C)	DBD	Linker length	HR-A/B (hydrophobic heptad repeats)	NLS (nuclear localization signal)	NES (nuclear export signal)	AHA (C-terminal activator)
***GhHsf7***	Gorai.003G183900	A1a	41-134	29	163-214	(248)KKRRLK	(501)LTEQMGLL	(457)FWEDLLV
***GhHsf4***	Gorai.003G053300	A1b	20-113	36	139-177	(224)KKRRLSR	(488)LTEQMGLR	(437)DVFWEQFL
***GhHsf25***	Gorai.008G225200	A1c	41-134	29	163-216	(248)KKRR	(497)LTEQMGLL	(450)VNSPFFHDLF
***GhHsf21***	Gorai.007G033300	A1d	42-135	29	164-207	(249)KKRR		(452)DSSSFWDDLI
***GhHsf10***	Gorai.004G257000	A1e	21-114	26	140-178	(225)KKRR	(489)LTEQMGLL	(437)DVFWEKFL
***GhHsf38***	Gorai.013G183500	A1f	21-114	23	137-184	(222)KKRRLHR		(401)DTFWEHFL
***GhHsf39***	Gorai.013G220400	A2	46-139	23	162-213	(243)RKRR	(369)LVDQMGYL	(320)ETLWEELVHEDL
***GhHsf17***	Gorai.006G158000	A3	47-140	20	160-191	(242)RMRRK		(365)PGYFISSPEDL (396)DVWSMDFDATV
***GhHsf34***	Gorai.011G036400	A4a	10-103	14	117-180	(205)RKRR	(390)LAEQMGHL	(339)DIFWEQFLTE
***GhHsf37***	Gorai.012G044200	A4b	10-103	18	121-175	(198)KKRK		(246)TLFLEIGETIG (372)GFWERFLTEV
***GhHsf32***	Gorai.010G240800	A4c	10-103	21	122-174	(205)RKRR	(371)LTEQMGHL	(319)DGFWEQFLTE
***GhHsf18***	Gorai.006G224000	A5a	20-113	17	130-185	(217)KKRR	(261)LRLELS	(358)SPSLTMMSQL (426)DVFWERFLTE
***GhHsf13***	Gorai.005G027500	A5b	21-114	12	126-189	(216)KKRR	(257)LRLELS	(375)SPILTRMSQP (443)DVFWEQFLTE
***GhHsf36***	Gorai.011G168400	A6a	38-131	30	161-202	(236)KKRQRR	(345)LVEQLRYL	(270)EVTELDKLVM (310)DEGFWDDLMDGDTH
***GhHsf19***	Gorai.006G242400	A6b	39-132	34	156-182/216-243	(238)KRRRR	(328)LVEQLGFL	(264)EVVELDGMVM (295)DEGFWNDLLND
***GhHsf8***	Gorai.004G076900	A7a	43-136	36	172-246	(242)KKRRR	(350)LADRLGYL	(219)NPAFLRQLM (318)DEGFWEELLNE
***GhHsf23***	Gorai.007G139600	A7b	40-133	32	165-230	(240)RKRMR		(217)NPSFLQQLM (320)DEGFWEELLNE
***GhHsf40***	Gorai.N013300	A8a	1-83	37	120-142	(188)KENNWR	(361)LTDQMGHL	(253)DFWMNIDFVKV (279)DDGAWEKLLL
***GhHsf24***	Gorai.008G170800	A8b	11-104	35	139-161	(103)RRK		(267)DFWMDIDFVKA (293)DDGAWEKLL
***GhHsf28***	Gorai.009G032300	A9a	113-206	24	230-269	(294)RLTKKRK	(418)IYLELEDL	(429)KQCSWGGFASEL
***GhHsf1***	Gorai.001G012700	A9b	80-173	22	195-240	(274)KKFKKRRR	(329)IYVELKQL	
***GhHsf12***	Gorai.004G284200	A9c	32-125	22	137-214	(253)RKKRR	(468)VYLELEDL	(479)KPSNLTGFVNDL
***GhHsf3***	Gorai.003G023500	B1a	6-99	53	152-195	(258)KKRAR		
***GhHsf22***	Gorai.007G053900	B1b	6-99	44	143-189	(257)KKR		
***GhHsf35***	Gorai.011G105700	B1c	6-99	46	145-184	(98)RRK		
***GhHsf20***	Gorai.007G010900	B2a	23-116	47	163-203	(257)KRAR		
***GhHsf6***	Gorai.003G160600	B2b	25-118	46	164-199	(262)KRVRR		
***GhHsf9***	Gorai.004G208800	B2c	24-117	60	167-200	(263)KRVRR		
***GhHsf26***	Gorai.008G244400	B2d	17-103	51	154-187	(248)KRLRK		
***GhHsf5***	Gorai.003G091700	B3	21-114	36	150-177	(216)RKRKRKR		
***GhHsf30***	Gorai.010G020700	B4a	18-104	74	178-212	(276)KKR	(305)IGLNLM	
***GhHsf29***	Gorai.009G213100	B4b	21-114	74	185-220	(325)KKR	(351)LRLNLM	
***GhHsf16***	Gorai.006G125000	B4c	17-110	38	148-182	(160)RLRRK		
***GhHsf14***	Gorai.005G102000	B4d	21-114	67	181-212	(193)KLRRR	(333)LGLNLM	
***GhHsf2***	Gorai.002G135200	B4e	21-114	57	171-204	(297)KKR	(324)LSCIYR	
***GhHsf33***	Gorai.011G027400	B5a	20-125	43	168-200	(182)RREK		
***GhHsf15***	Gorai.006G087100	B5b	22-123	16	139-180	(105)KHEKFKR		
***GhHsf31***	Gorai.010G070900	C1a	9-102	15	117-157	(188)KKRR		
***GhHsf11***	Gorai.004G280100	C1b	9-102	29	131-163	(198)KRR		
***GhHsf27***	Gorai.009G024700	C1c	8-101	10	111-163	(194)KKRR		

The HR-A/B domain, composed of several hydrophobic heptad repeats, is responsible for the interactions with Hsfs to generate Hsfs dimers or trimers through a helical coiled-coil structure. Similar to other plant Hsf proteins, cotton class B Hsf proteins are compact without an insertion between HR-A and HR-B (Table 2, Figure 2). Class A Hsf proteins have an insertion of 21 amino acids between the HR-A and HR-B regions, and seven amino acid insertions were found in class C between the HR-A and HR-B regions. GhHsf7(A1a) has the typical HR-A/B structure, consisting of L × (6aa)L × (6aa)L: RQQQ--21aa--QQ: MMSFLAK. In contrast with other reported class C proteins, the structure between HR-A and HR-B in GhHsfs has its own characteristic. The structure in GhHsf27(C1c) is L × (6aa) L × (6aa): MNKRLE(A/T) (A/T)--4aa--QQ: MMAFLY, indicating that cotton class C proteins were probably variable during evolution. Based on the characteristics of HR-A/B, we divided 40 GhHsfs proteins into classes A (n = 22), B (n = 15) and C (n = 3). The oligomerization domain of the HR-A/B region is a conserved domain close to the DBD and separated by a flexible linker. Linkers of 12 to 37 amino acid residues exist in class A, 16 to 77 residues in class B and 10 to 29 residues in class C, with the longest average linker length in class B and the shortest in class C. In class A and class C, the variable length of the linker between the DBD and the HR-A/B region offers additional support for this classification.

Two clusters of basic amino acid residues (K/R motifs) found in Hsf proteins may contribute to their dynamic intracellular distribution between the nucleus and cytoplasm [14, 53]. Pfam was searched for potential NLS and NES domains in cotton Hsf proteins. As expected, all Hsfs proteins contain K/R motifs (1 to 5 repeats) (Table 2). This indicates that GhHsfs proteins are located in the cell nucleus. Hsfs subcellular localizations are also affected by the NES. NES is a leucine-rich motif at the C-terminus required for the NES receptor-mediated nuclear export [29]. Pfam searches showed that 16 class A cotton Hsfs contain the NES signal peptide—LTEQMGLL, while the NES in class B Hsfs is typically L(G/R)LNLM.

The function of class A Hsfs as a transcription activator is mediated by short activator peptide motifs (AHA motifs) located in the C-terminal domains [54]. Previous studies have shown that AHA motifs are characterized by aromatic (W, F, Y), large hydrophobic (L, I, V) or acidic (E, D) amino acid residues. Similar to other class A Hsf proteins, all A-type GhHsfs contain an AHA motif except GhHsf1. The length of AHA motifs in 21 GhHsfs are variable and rich in F, W and D amino acid residues. The C-terminal of GhHsf1 (A9b) does not contain a typical AHA motif but includes a distinct pattern of tryptophan residues, which probably contributes to the activator function. *In vitro* pull-down assays have shown that AtHsfA8 is inactive in yeast monohybrid assays and it does not recruit any components of the transcription machinery [21]. This indicates that cotton HsfA9b does not regulate gene expression independently at the transcriptional level.

### Phylogenetic analysis of the cotton Hsf family and Hsf gene duplication in the D-subgenome

**Figure 4:**
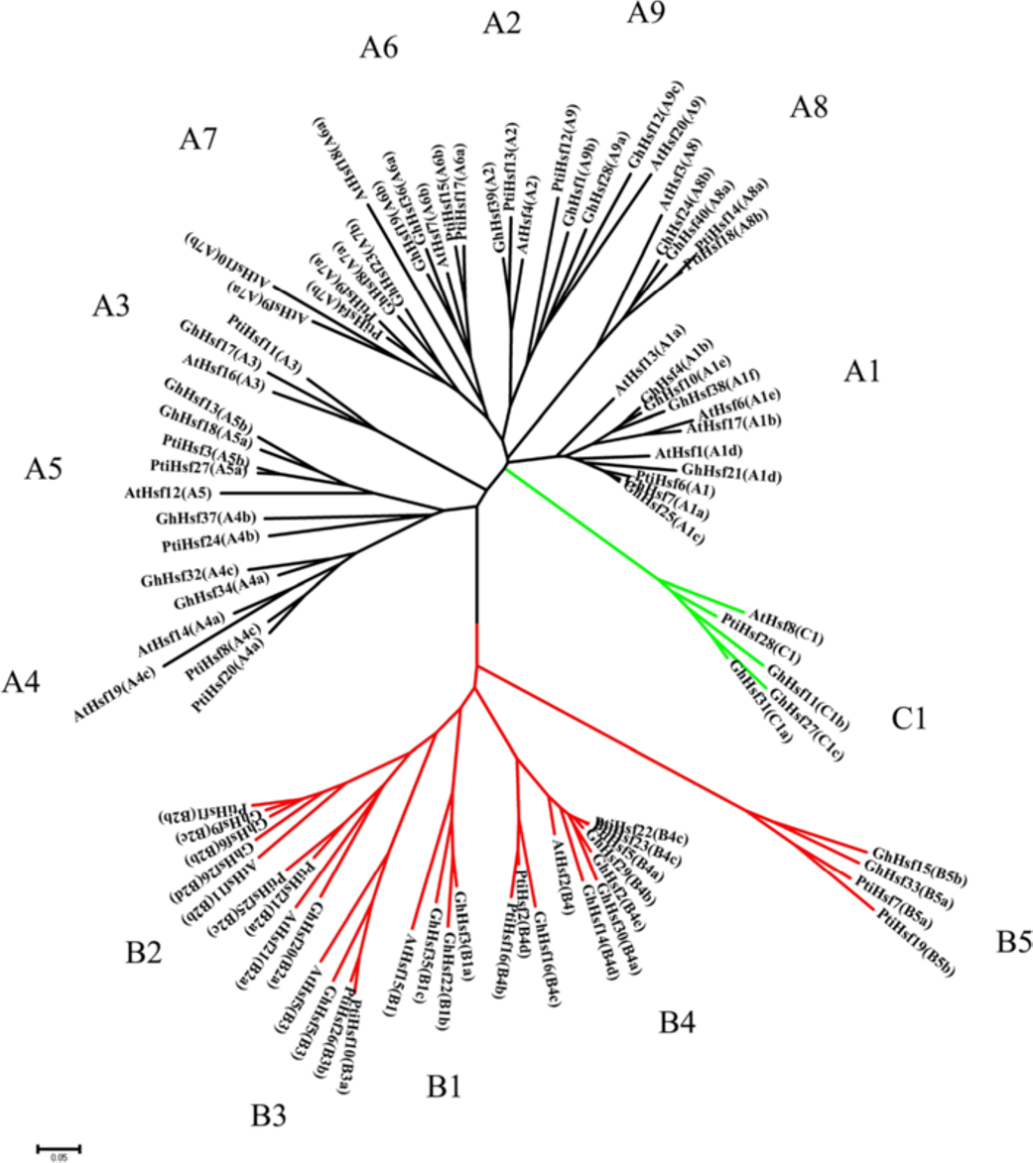
**Neighbor-joining phylogeny of Hsfs from** ***G. hirsutum*** **,** ***P. trichocarpa*** **and** ***A. thaliana*** **.** The phylogenetic tree was obtained using the MEGA 5.0 software on the basis of amino acid sequences of the conserved domains of Hsfs including the DNA-binding domain, the HRA/B region and other conserved domains.

In order to analyze the evolution of *Hsf*s, 28 *Populus trichocarpa* Hsfs (PtiHsfs), 21 *Arabidopsis thaliana* Hsfs (AtHsfs) and 40 *Gossypium hirsutum* Hsfs (GhHsfs) were used to generate an unrooted phylogenetic tree. Genome sequencing revealed that *Populus trichocarpa* is evolutionarily closest to Upland cotton; therefore, a phylogenetic tree was constructed based on the cotton Hsf proteins. As shown in Figure 4, Hsf proteins from *G. hirsutum, Arabidopsis thaliana and Populus trichocarpa* were clearly grouped into three different classes (A, B and C). Class A is composed of 22 Hsf proteins, which were then grouped into nine distinct sub-clades (A1–A9). The C-type Hsfs from the three plant species also constituted one distinct clade which appeared more closely related to the Hsf A-group. Correspondingly, the B-type Hsfs from the three plant species was grouped into a separate clade. Class B was classified into five subgroups and class C had only three members. As expected, the duplicated cotton *Hsf*s clustered on the same group.

**Figure 5:**
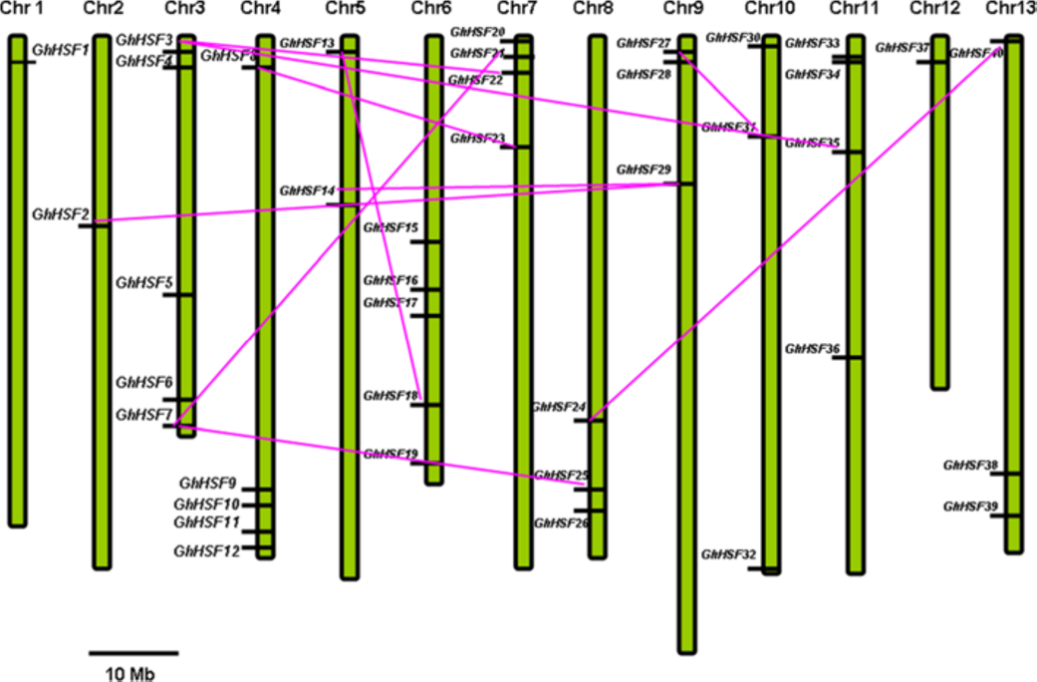
**Localization and duplication of** ***GhHsf*** **genes in the cotton genome.** Forty *Hsfs* were mapped on different chromosomes in the cotton D-subgenome using the alignment software. The chromosome numbers are indicated above and segmental duplications are joined by lines. Scale bar: 10 Mbp.

ClustalW was used to analyze the duplication events that may have occurred during the evolution of the cotton genome. Ten duplicated gene pairs of the 40 cotton *Hsf* genes were identified between chromosomes, no duplication events within the same chromosome (Figure 5). Chromosome 7 contained the most duplication events, while chromosome 1, 12, 13 were not involved in any duplication events. *GhHsf3* and *GhHsf7* participated in two duplication events. *GhHsf3* was duplicated with *GhHsf35* and *GhHsf22*, *GhHsf7* with *GhHsf21* and *GhHsf25*. Class A proteins contained five duplication events, class B contained four and class C contained one. These results indicate that single gene duplication events are responsible for the expansion of the *Hsf* gene family in cotton.

### Gene structure and mutation analysis of cotton Hsfs compared with G. hirsutum and G. raimondii

In plants, most genes are interrupted genes with one or more exons and several introns. The arrangement of intron and exon localization can be used to analyze the evolutionary relationships among different gene members. In order to analyze the gene structures, all the *GhHsf* genes were compared with the genomic sequence of *G. raimondii*. The results showed that 38 *Hsf* genes contain one intron, and only two *Hsf* genes contain two introns (Table 3). No intronless *Hsf*s were found in the cotton genome. Most *GhHsf* genes that were clustered in the same subfamilies shared strikingly similar exon-intron structures. For example, *GhHsf* genes in class A1 contain one intron and two exons. The intron patterns, which correlate well with the phylogentic clades, strongly support the existence of close evolutionary relationships within the same subfamily. The intron-exon patterns of nine duplicated genes on different chromosomes were also conserved, with the exception of the duplication pair between *GhHsf2* and *GhHsf29*. One intron was inserted in *GhHsf2* on the chr2 origin of *GhHsf29* (Chr9) during evolution. Previous reports have shown that the conservation of exon-intron structure in subfamilies of paralogous genes exists in the maize WRKY transcription factor family [55]. This characteristic in the same subfamily is important for gene divergence. The different gene structure associated with the different subfamilies may be the result of gene expansion from ancient paralogs or multiple origins of gene ancestry.

**Table 3:** **Analysis of** ***GhHsfs*** **exon-intron structures**

Gene	Locus in ***G.ramondii***	Exon 1	Intron 1 (bp)	Exon 2	Intron 2 (bp)	Exon 3
*GhHsf1*	Gorai.001G012700	32---457	593	458---1063		
*GhHsf2*	Gorai.002G135200	1---249	85	250---974	306	975---999
*GhHsf3*	Gorai.003G023500	50---253	625	254---937		
*GhHsf4*	Gorai.003G053300	136---381	1414	382---1644		
*GhHsf5*	Gorai.003G091700	1---249	1268	250---717		
*GhHsf6*	Gorai.003G160600	147---407	74	408---1079		
*GhHsf7*	Gorai.003G183900	103---411	1314	412---1650		
*GhHsf8*	Gorai.004G076900	154---468	706	469---1242		
*GhHsf9*	Gorai.004G208800	92---349	120	350---1027		
*GhHsf10*	Gorai.004G257000	1---249	1472	250---1512		
*GhHsf11*	Gorai.004G280100	1---213	90	214---984		
*GhHsf12*	Gorai.004G284200	1---282	89	283---1091	84	1092---1491
*GhHsf13*	Gorai.005G027500	15---263	597	264---1502		
*GhHsf14*	Gorai.005G102000	1---249	151	250---1032		
*GhHsf15*	Gorai.006G087100	1---276	2513	277---576		
*GhHsf16*	Gorai.006G125000	1---237	127	238---777		
*GhHsf17*	Gorai.006G158000	177---503	744	504---1625		
*GhHsf18*	Gorai.006G224000	71---316	607	317---1504		
*GhHsf19*	Gorai.006G242400	1---303	79	304---1023		
*GhHsf20*	Gorai.007G010900	148---402	104	403---1128		
*GhHsf21*	Gorai.007G033300	77---388	2621	389---1516		
*GhHsf22*	Gorai.007G053900	1---366	1399	367---1050		
*GhHsf23*	Gorai.007G139600	1---306	541	307---1038		
*GhHsf24*	Gorai.008G170800	74---292	2270	293---1147		
*GhHsf25*	Gorai.008G225200	282---590	2770	591---1817		
*GhHsf26*	Gorai.008G244400	169---384	111	385---1083		
*GhHsf27*	Gorai.009G024700	85---294	76	295---1136		
*GhHsf28*	Gorai.009G032300	1---525	102	526---1344		
*GhHsf29*	Gorai.009G213100	25---273	123	274---1107		
*GhHsf30*	Gorai.010G020700	1---219	122	220---942		
*GhHsf31*	Gorai.010G070900	171---383	82	384---1193		
*GhHsf32*	Gorai.010G240800	77---292	83	293---1231		
*GhHsf33*	Gorai.011G027400	1---282	1220	283---666		
*GhHsf34*	Gorai.011G036400	1---216	97	217---1212		
*GhHsf35*	Gorai.011G105700	280---483	298	484---1119		
*GhHsf36*	Gorai.011G168400	1---300	511	301---1074		
*GhHsf37*	Gorai.012G044200	234---449	172	450---1418		
*GhHsf38*	Gorai.013G183500	213---461	1886	462---1511		
*GhHsf39*	Gorai.013G220400	33---356	85	357---1175		
*GhHsf40*	Gorai.N013300	8---226	1504	227---1210		

The nucleotides in the coding regions of *GhHsfs* and *GrHsfs* were compared to analyze the mutation frequency of all the *Hsf* genes. The mutation frequency of *Hsf* genes is 0.00996 during the evolution from *G. raimondii* to *G. hirsutum*. The synonymous and non-synonymous substitutions are 0.00433 and 0.00564, respectively. This indicates that the rate of nucleotide substitution has increased in allotetraploid genomes relative to the diploids, and that the rate of non-synonymous substitutions is higher than that of synonymous substitutions. This result is consistent with the molecular evolutionary analyses of protein-coding regions at the genome level.

### Protein localization analysis of cotton Hsf proteins

**Figure 6:**
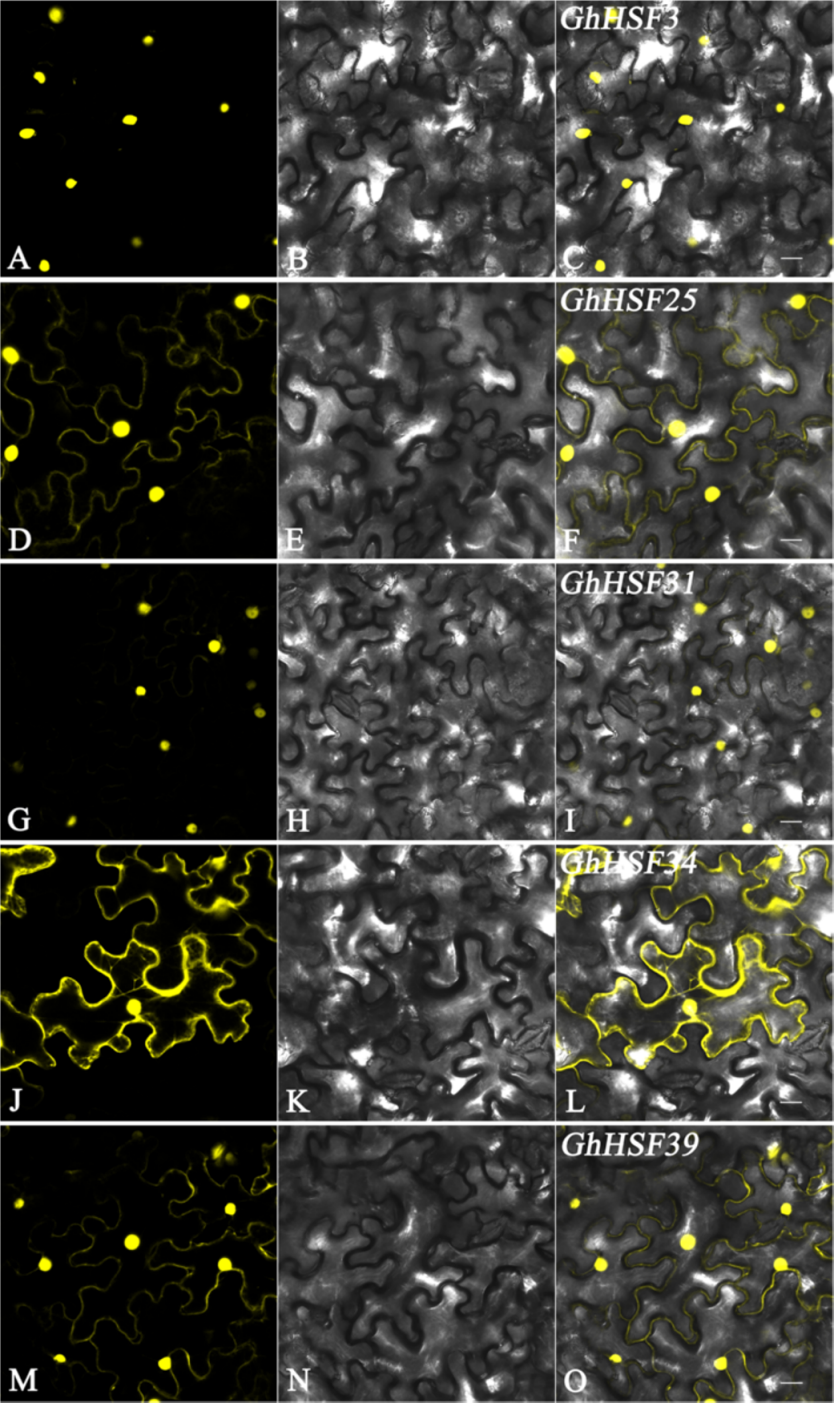
**GhHsf proteins’ subcellular localization analysis. A**, **D**, **J**, **G**, **M** (left column), black-field images; **B**, **E**, **H**, **K**, **N** (middle column), bright-field images; **C**, **F**, **I**, **L**, **O** (right column) merged images. Scale bar: 25 μm.

In order to investigate the subcellular localization of cotton Hsfs, five genes (*GhHsf3, 25, 31, 34* and *39*) from three classes were chosen to generate GFP fusion constructs (*pBIB-35S::GhHsfs-GFP::NOS*). The constructs were introduced into *Agrobacterium EHA105* and infiltrated into tobacco leaf cells for protein localization analysis by confocal laser scanning microscopy. Three types of localization were identified among the five Hsfs (Figure 6). GhHsf3 (B1a) and GhHsf31 (C1a) were strongly expressed only in the nucleolus. GhHsf25 (A1c) and GhHsf39 (A2), which contain NES motifs, were strongly expressed in the plasma membrane and nucleolus. GhHsf34 (A4a) was expressed in the plasma membrane, nucleolus and cytoplasm, and was also observed in the scaffold. The localization of GhHsf proteins is consistent with their protein structure [53, 56, 57]; that is, GhHsf25, 34 and 39 have NES motifs, while GhHsf3 and GhHsf31 do not.

The effect of heat shock on protein expression was analyzed. At 3 days after infection with the GFP fusion constructs, tobacco plants were treated with heat stress for 1 h and then transferred to normal conditions. The GFP signals of all five Hsf fusions were enhanced significantly after heat treatment (Additional file 4: Figure S1). This result revealed that protein expression levels were also enhanced after heat shock, and that different cotton Hsf proteins may perform different roles in stress tolerance associated signal transduction during heat stress.

### Expression profiles of cotton Hsf genes in different tissues

**Figure 7:**
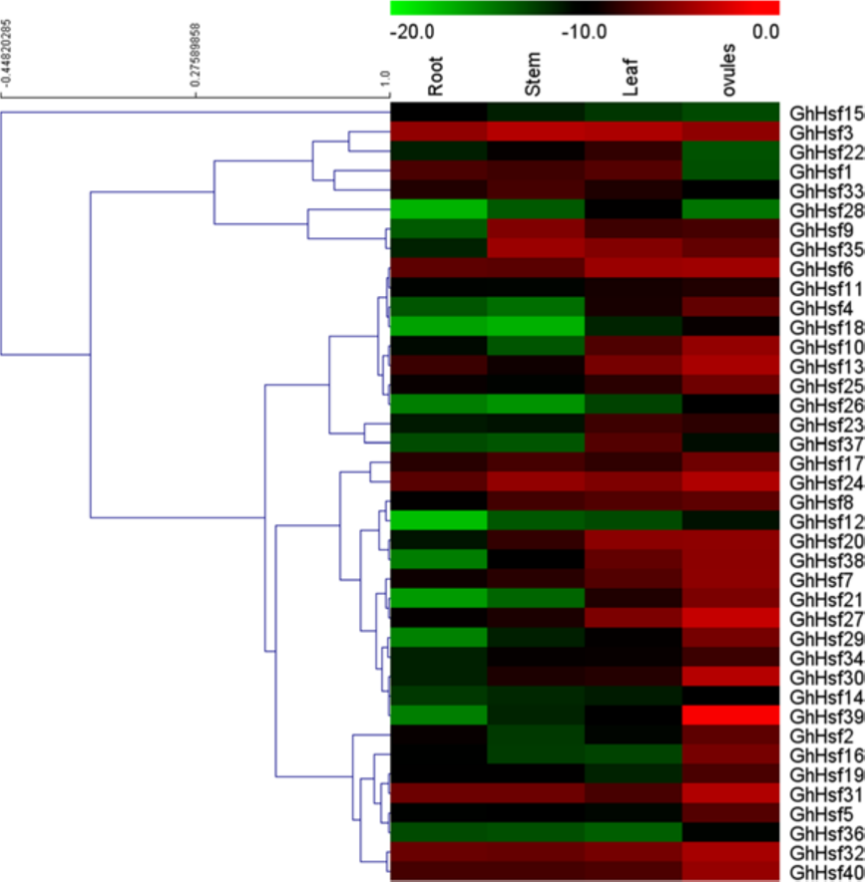
**Expression analysis of** ***GhHsf*** **s in different tissues.** Quantitative RT-PCR analysis of the expression level of *GhHsfs* in different tissues or organs including roots, stems, leaves and developing ovules. Results were normalized using ubiquitin gene expression as the internal control.

Analysis of the tissues expression profiles of *GhHsfs* by qRT-PCR showed that most *GhHsfs* were expressed in all the tested tissues including root, stem, leaf and ovules. All of *GhHsf* genes were highly expressed in the leaves, and none of the genes exhibited restricted expression in a single tissue (Figure 7). Interestingly, most of *GhHsf* genes were expressed at very low levels in the root, with the exception of *GhHsf31, 32*, the expression of which was approximately three times higher in the root than that in other tissues. In addition, analysis of the digital data showed similar expression of duplicated genes located on different chromosomes, such as *GhHsf2* and *GhHsf29*. Both of these genes exhibited highest expression in the ovules and lowest in other tissues.

**Figure 8:**
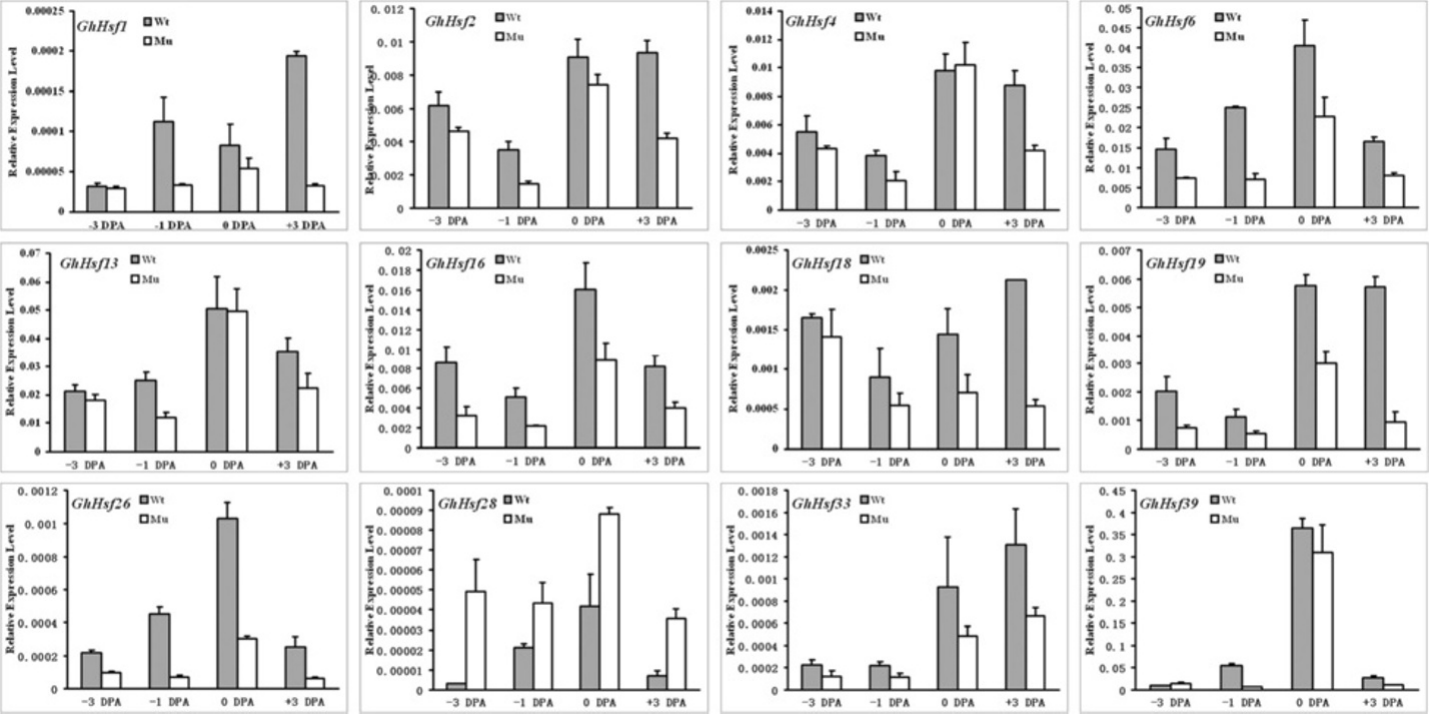
**Real-time quantitative PCR analysis of** ***GhHsfs*** **in Xu-142 and its fiberless mutant, XU142fl.** Quantitative RT-PCR was performed to analyze the expression of *GhHsfs* in Xu-142 (*Gossypium hirsutum*) WT ovules and XU142fl (*fuzless-lintless mutant* of Xu-142) ovules. -3DPA, -1DPA, 0DPA and +3DPA represent the ovules at different days post anthesis. Results were normalized using ubiquitin gene expression as the internal control(error bars indicate standard deviation).

The accumulation of reactive oxygen species (ROS) is implicated in cotton fiber development [58]. To investigate the involvement of *GhHsfs* in the cotton fiber development, a comprehensive analysis of their expression was performed in a WT (Xu-142) and fiberless mutant Xu-142 fl. The results showed that most genes had no difference of expression in a comparison of Xu-142 and the fl mutant, with the exception of *GhHsf1, 2, 4, 6, 13, 16, 18, 19, 26, 28, 33 and 39* (Figure 8). Among these twelve genes, the most significant difference between Xu142 and fl mutant was observed for *GhHsf1*, with approximately six times greater expression in the WT during fiber initiation (from -3DPA to 3 DPA) compared with that in the fl mutant. In terms of the abundance of gene expression during fiber initiation, the most abundant expression of *GhHsf39* was more than 1000-times greater than that of *GhHsf1,* indicating that GhHsf39 may act as an important role like recovering oxidative stress or development signal etc. during fiber initiation

### Expression analysis of cotton Hsf genes under heat shock

**Figure 9:**
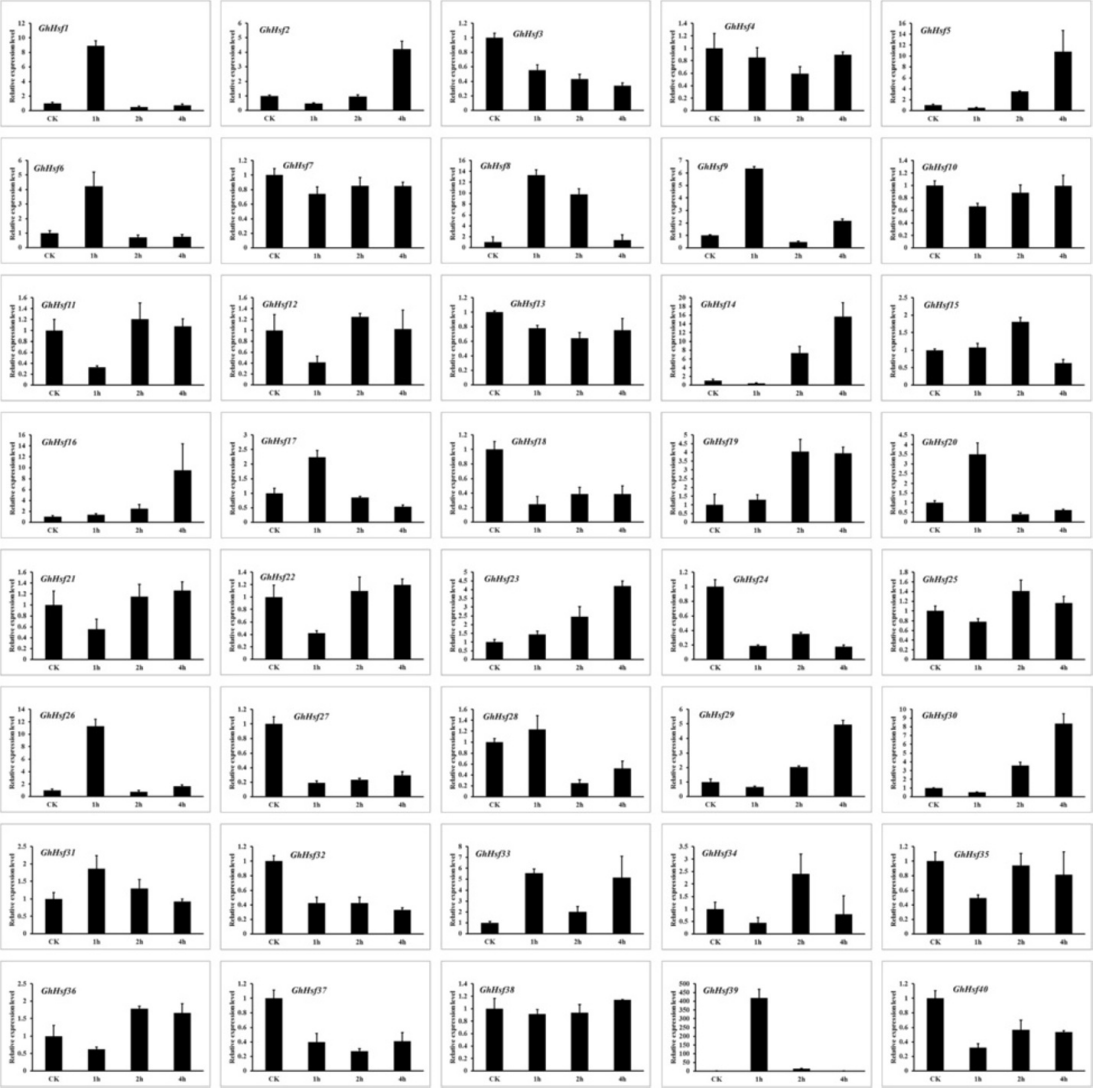
**Expression analysis of** ***GhHsfs*** **in developing leaves during heat stress.** Quantitative RT-PCR was performed to analyze the expression level of *GhHsfs* during heat stresses in young leaves (the third leaf of 20-day-old seedlings). The vertical axis represents the fold-change in expression relative to CK (1-fold). The horizontal axis represents the different times of treatment. CK: untreated young leaf; 1 h: young leaves were subjected to 42°C for 1 h. 2 h, 4 h: young leaves were returned to normal growth conditions and recovered for 2 h and 4 h after heat stress. Results were normalized using ubiquitin gene expression as the internal control (error bars indicate standard deviation).

The expression patterns of cotton *Hsf* genes during heat stress were analyzed by qRT-PCR. Three patterns of expression were observed among the cotton *Hsf* gene families after heat treatment for 1 h, followed by recovery for 2 to 3 h (Figure 9). The gene expression patterns of *GhHsf4*, *7*, *10*, *25* and *38* were not changed significantly. However, the gene expression patterns of *GhHsf3*, *13*, *18*, *21*, *22*, *24*, *27*, *32*, *35*, *37* and *40* were inhibited after heat treatment, while those of the remaining genes were strongly up-regulated. The up-regulated genes were assigned to two categories according to the time at which the increase occurred. The expression of *GhHsf1*, *6*, *8*, *9*, *17*, *20*, *26* and *39* increased instantly in response to heat treatment, and decreased quickly during the recovery process. The highest increase (400-fold) of was observed for *GhHsf39*. The other 16 genes (*GhHsf2, 5, 11, 12, 14-16, 19, 23, 28-31, 33, 34 and 36*) were inhibited after heat treatment, and their expression levels slowly increased during the recovery process. These results provide an essential clue to the functional diversification of several Hsfs such as GhHsf1, 6 and 8 as the part of heat stress signaling system, while GhHsf2, 5, 11 and 12 proteins play critical roles in protein refolding.

## Discussion

### Cotton contains the highest number of Hsf family members among the sequenced plant genomes

Upland cotton (*G. hirsutum*) is an important commercial cotton species, accounting for approximately 95% of all cotton production worldwide. Upland cotton originated from A-genome diploids native to Africa and D-genome diploids such as *G. raimondii* native to Mexico diverged about 5 to 10 million years ago. These two genomes were then reunited approximately 1 to 2 million years ago and generated tetrapolid Upland cotton (2n = 4X = AADD) [41, 59]. Due to the high similarities in the gene sequence and genome organization between the A and D genomes, the publication of the D-genome sequence provides a useful tool to analysis gene function in Upland cotton. In this study, we cloned and analyzed 40 *Hsf* genes from the Upland cotton D-subgenome including 22 class A, 15 class B and three class C members.

Previous studies have indicated that the increase in the number of transcriptional genes is an important event during the evolution of complex plant systems. It is hard to achieve the expansion of transcriptional regulating genes through single gene duplications alone, indicating the importance of genome duplications in the process of gene expansion. It was estimated that more than 90% of the increase in transcriptional regulating genes over the last 150 million years results from genome duplication in the *Arabidopsis* lineage. Comparison of the *Hsf*s in the D-subgenome of Upland cotton with predicted genes in the *G. raimondii* genome indicates that there is no gene-loss during tetrapolid Upland generation. As tetrapliod cotton with an “A” and “D” subgenome, Upland cotton contains at least 80 *Hsf* genes that originate from an ancestral polyploidy event about 2 million years ago [41, 59].

The D-subgenome contains twice as many members as *Arabidopsis* and four times as many members in Upland cotton due to the genome polyploidation. In addition, 10 duplications were found between different chromosomes among three different *Hsf* gene classes, duplications in cotton being of the segment type rather than tandem gene or cluster duplication. Gene duplication among both different chromosomes and subgenomes has contributed to the *Hsf* family being the largest among the reported plant genomes.

Evolutionary analyses of protein-coding regions demonstrated that the rate of nucleotide substitution has increased in allotetraploid genomes relative to the diploids. The ratio of nonsynonymous substitutions is higher than those of synonymous mutations [60]. Nucleotide substitutions in the *Hsf* gene family in Upland cotton also follows this rule, indicating that mutations in the *Hsf* genes are increased under artificial selection. The total mutation frequency of *Hsf* genes (0.00996) is slightly lower than the frequency of whole genome mutations from *G. raimondii* to *G. hirsutum* [41, 60], supporting the view that *Hsf* is a highly conservative gene family.

Previous reports have indicated that there are 406 *myb* genes in *G. raimondii* compared with 163 genes in the *Arabidopsis* MYB superfamily. Furthermore, the subgroup 9 MYB family has six members known only in cotton, comprising a possible ‘fiber clade’ distinct from the *Arabidopsis thaliana* GL1-like subgroup 15, which is involved in trichome and root hair initiation and development [41]. Similar to the unique MYB clade, the Upland cotton D-genome contains three *Hsf* family members including *HsfB4e, HsfA1f, HsfA9c*, which are unreported in other organisms. *HsfA9* is a specialized *Hsf* of embryogenesis and seed maturation, which represents functional diversification in the *Hsf* family [61]. *HsfA9* is known to be controlled by transcription factor ABI3 (abscisic acid-insensitive 3) in *Arabidopsis* [62]. Ectopic expression of *HsfA9* resulted in up-regulation of heat shock protein (Hsp) expression and Hsp101 accumulated in leaves under unstressed conditions, while over-expression of sunflower *HsfA9* in tobacco seeds improved seed longevity through Hsp accumulation. Therefore, it can be speculated that HsfA9 controls seed development and longevity through interactions with other proteins such as DREB2 or ABI3. In cotton, there are three different HsfA9 members with unique HsfA9C (GhHsf11). *GhHsf11* and *GhHsf1* (assigned to *HsfA9c* and *HsfA9b*, respectively) are upregulated in the recovery process after heat shock and are also strongly induced after double fertilization. Upland cotton is vulnerable to heat stress because cotton begins to flower in summer. The impact of heat stress on cotton is to delay crop maturity and reduce overall lint yields and quality. These two genes probably have unique roles of reducing heat stress injury during summer bolling development.

### GhHsf39 is an early heat shock response gene

Heat stress causes water deficit, leaf senescence and even male infertility in cotton, and it becomes a serious handicap for cotton production [63]. Hsfs are the major regulators of heat shock protein transcription in plants responding to cellular stresses like increased temperature. The functional diversification within the cotton *Hsf* gene family was investigated by qRT-PCR analysis of the expression patterns under heat stress. Three distinct patterns of expression in response to heat shock were observed among the *Hsf* gene family investigated. In the rapid-response type, the *Hsf* genes expression levels were instantly and markedly upregulated after heat shock and decreased quickly when heat stress was lifted. Eight of the *Hsf* genes responded according to this type, with expression levels that were increased to more than 10 times those in untreated plants. Among these genes, *GhHsf39*(A2) is a typical member, the transcripts of which were enhanced by approximately 400 times in 1 h after heat stress. HsfA2 is considered a key regulator of heat tolerance in tomato and *Arabidopsis* due to its high activation of Hsp gene transcription and its continued accumulation during heat stress. *Arabidopsis* HsfA2 is localized in the nucleus and regulated by itself and HsfA1a–e [14, 56]. According to the expression changes observed during heat stress, we deduced that *GhHsf39* has a similar function to *HsfA2* in tomato and *Arabidopsis*. In contrast to *Arabidopsis*, class B2 *Hsf* genes in cotton, including *GhHsf 20*, *6*, *9* and *26* (B2a–B2d), respond rapidly to heat stress. Domain analysis shows that the Hsfs in class B lack the AHA activator domain and it is possible that these proteins serve as transcriptional coactivators with class A Hsfs, although the functional roles of these four class B Hsfs in cotton require further investigation. In the later-response type, the *Hsf* gene expression levels were not instantly upregulated or inhibited after heat shock, but slowly and continuously increased during plant recovery from cell damage. These results indicated that different types of GhHsfs probably have different roles in protein refolding under abiotic stresses.

### Hsf proteins act as ROS regulators during fiber development

Previous studies have shown that hydrogen peroxide (H_2_O_2_) and other ROS serve as developmental signals for the onset of secondary wall differentiation [64, 65]. H_2_O_2_ and other ROS at appropriate concentrations are also required for cell elongation probably through cleaving polysaccharides to relax the cell wall [66, 67]. Many genes, such as *GhAPX1*, involved in modulating ROS concentrations are upregulated at both the transcriptional and translational levels in cotton. GhAPX1 is implicated in detoxification of H_2_O_2_ produced by quick-fiber elongation, which is supported by the observation that enhanced transcript abundance and enzymatic activity of GhAPX1 during fiber elongation as well as fiber length can be improved by exogenous H_2_O_2_ [58]. The observation that the H_2_O_2-_scavenger activity associated with the *APX2* gene can be regulated directly by Hsf class A has been confirmed in *Arabidopsis*. In this study, 28 *Hsf* genes were strongly expressed during ovule and fiber development. Moreover, 12 genes, including *GhHsf1*, *6*, *16*, *19*, *33* and *39* exhibited significant differential expression during fiber initiation in Xu142 and its corresponding fl mutant. Among these genes, the most significant difference between Xu142 and fl mutant existed in *GhHsf1*, the expression of which was approximately six times greater in the WT than in the fl mutant during fiber initiation (from -3 DPA to 3 DPA). During fiber initiation, *GhHsf39* was expressed most abundantly at more than 1000-times the levels of *GhHsf1,* indicating a predominant role for GhHsf39 in this process. Transcriptomic and proteomic studies have confirmed high expression of several Hsps at the stages of fiber initiation and elongation. Although the hypothesis that GhHsf regulates these Hsps directly during fiber development needs to be confirmed in detail, our results indicate that Hsf proteins act as ROS regulators during fiber development.

## Conclusions

The complexity of the Hsf family has been the subject of many investigations in different plant species. In this study, 40 full-length *Hsf* genes were identified in the cotton genome. Based on the structural characteristics of the proteins and comparison with homologues from other species, the 40 *GhHsf*s were grouped into three different classes. Segmental and tandem duplications were examined and shown to contribute to the expansion of the *Hsf* family in the cotton genome. The expression profiles in different tissues at different developmental stages as well as in leaves exposed to high temperature indicated that GhHsfs play a role in different aspects of cotton abiotic stress tolerance and fiber development.

## Electronic supplementary material

Additional file 1: Table S1: Primers used in this study. (XLS 33 KB)

Additional file 2: Table S2: The consensus position of *GhHsf* genes and the diploid genome (*G. arboretum* or *G. ramondii*). (XLS 20 KB)

Additional file 3: **The** ***GhHsf*** **gene sequences and the ORFs of** ***GaHsf*** **and** ***GrHsf*** **from** ***G. arboretum*** **and** ***G. ramondii*** [41, 44]. (TXT 141 KB)

Additional file 4: Figure S1: GhHsf proteins’ subcellular localization analysis after heat shock. A, C (left column) are the merged confocal images of GhHsf34-promoter-ORF-GFP and GhHsf39-promoter-ORF-GFP respectively before heat shock; B, D (right column) are the merged confocal images of GhHsf34-promoter-ORF-GFP and GhHsf39-promoter-ORF-GFP respectively after heat shock. Scale bar: 25 μm. (TIFF 1 MB)

## Abbreviations

Hsf: Heat shock transcriptional factor
DBD: DNA-binding domain
HR-A/B: Adjacent bipartite oligomerization domain
AHA: Activator motif
NLS: Nuclear localization signal
NES: Nuclear export signal
CTAD: C-terminal activation domain
HSE: Heat shock element
qRT-PCR: Quantitative real-time PCR.
