# Supplementary figures and images for: Genome-wide cloning, identification, classification and functional analysis of cotton heat shock transcription factors in cotton (Gossypium hirsutum)
Source: BMC Genomics. 2014 Nov 6;15(1):961. doi: 10.1186/1471-2164-15-961 (PMC4233062; doi:10.1186/1471-2164-15-961)

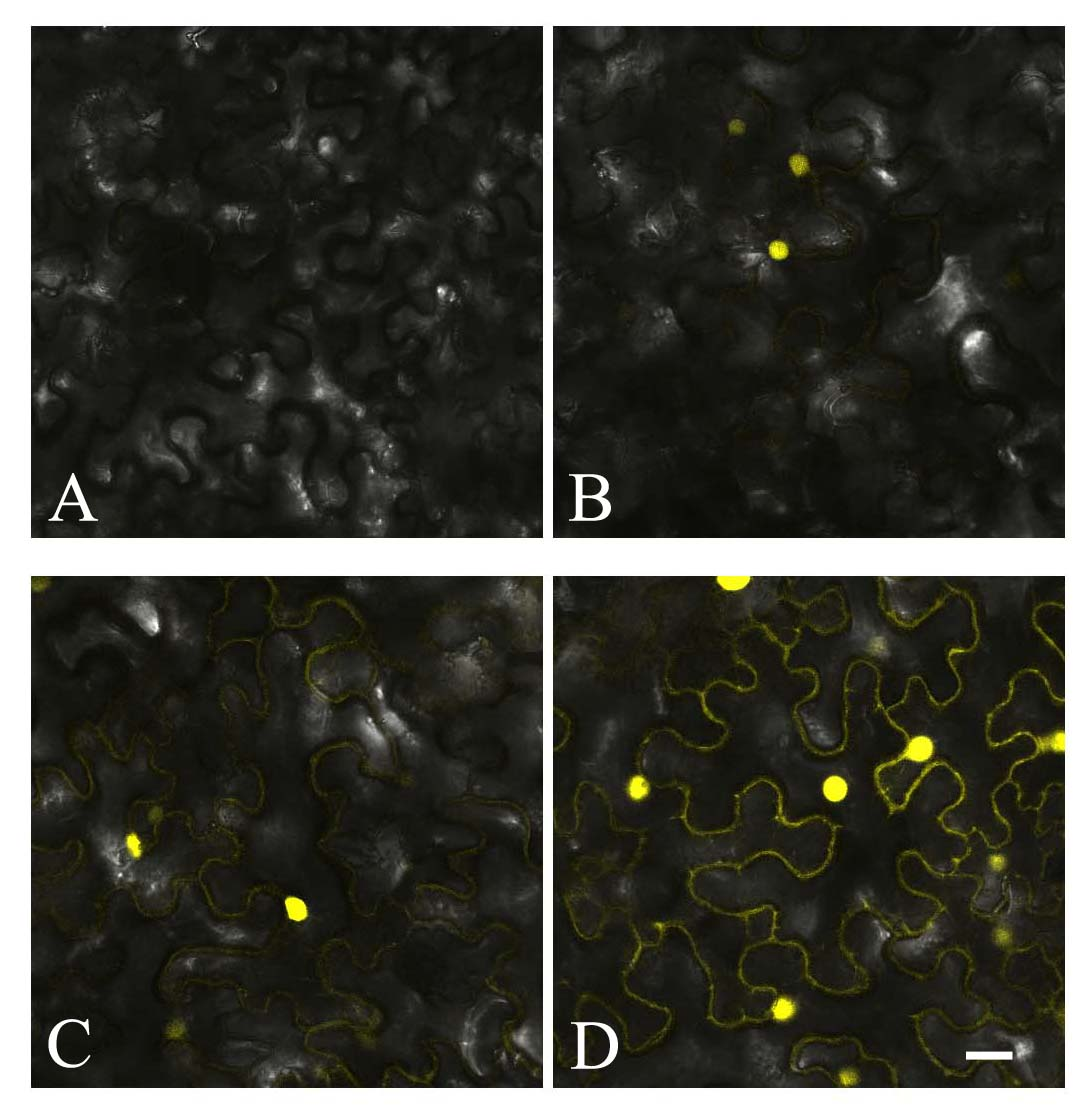

Supplement: Supplementary file 4 — Additional file 4: Figure S1: GhHsf proteins’ subcellular localization analysis after heat shock. A, C (left column) are the merged confocal images of GhHsf34-promoter-ORF-GFP and GhHsf39-promoter-ORF-GFP respectively before heat shock; B, D (right column) are the merged confocal images of GhHsf34-promoter-ORF-GFP and GhHsf39-promoter-ORF-GFP respectively after heat shock. Scale bar: 25 μm. (TIFF 1 MB) [file 12864_2013_6657_MOESM4_ESM.tiff]
